# Association between NNNS-II Profiles and Pharmacological Treatment in Infants with Prenatal Opioid Exposure

**DOI:** 10.21203/rs.3.rs-8099613/v1

**Published:** 2025-11-26

**Authors:** Barry Lester, Macie Donahue, Madison Ramirez Heil, Marie Camerota, Lynne Dansereau, Elisabeth Conradt

**Affiliations:** Brown University; The Warren Alpert Medical School of Brown University; Warren Alpert Medical School of Brown University; Warren Alpert Medical School of Brown University; Brown Alpert Medical School and Women and Infants Hospital, Providence

## Abstract

**Objective:**

Examine whether neonatal neurobehavioral profiles are related to need for pharmacological treatment among infants with prenatal opioid exposure.

**Study Design:**

Prospective cohort study of 217 infants with need for treatment determined using the Finnegan Neonatal Abstinence Tool (FNAST), Neonatal Withdrawal Inventory (NWI), or Eat Sleep Console (ESC). Neurobehavior was assessed with the NeoNatal Neurobehavioral Scale II (NNNS-II). Latent Profile Analysis (LPA) classified infants into neurobehavioral profiles and logistic regression assessed the association between NNNS-II profiles and need for treatment.

**Results:**

A 3-profile LPA solution best fit the NNNS-II data comprised of typical (67%), hyper-aroused (19%) and hypo-aroused groups (15%). Infants with atypical NNNS-II profiles were more likely to receive treatment (OR=3.45, 95% CI 1.21–9.81) compared to infants with typical profiles (*p* < .05, Table 4).

**Conclusion:**

Newborn neurobehavioral profiles may aid in early identification of infants requiring pharmacological treatment for opioid withdrawal, reducing length of stay and healthcare costs.

## Introduction

Neonatal opioid withdrawal syndrome (NOWS) is characterized by dysregulation of the central, autonomic, and gastrointestinal systems in some newborns with prenatal opioid exposure [1]. Symptoms of NOWS include a range of clinical signs including high pitched cries, increased muscle tone, tremors, poor feeding, and poor sleep [2, 3]. From 2010 to 2017, both the rate of maternal opioid-related diagnoses and the incidence of NOWS increased nationally [5–7]. Maternal opioid-related disorders increased from 3.5 to 8.2 per 1,000 delivery hospitalizations and the rate of NOWS increased from 4.0 to 7.3 per 1,000 birth hospitalizations [6]. The clinical presentation of NOWS varies depending on factors such as opioid type, maternal drug history, and maternal and infant metabolism [4]. Some infants exhibit only mild symptoms of NOWS, while others experience more severe symptoms requiring pharmacological treatments.

Typical hospital practices involve continuous monitoring of infants’ withdrawal symptoms for 3 to 7 days after birth, a practice that is costly and can impact care [4]. First-line treatment for NOWS typically includes non-pharmacological interventions which promote mother-infant bonding, including maternal rooming-in, skin-to-skin contact, and a low stimulation environment with reduced noise and light [2, 8]. For infants exceeding predefined symptom thresholds as measured by NOWS assessment tools, pharmacological treatments such as morphine are used to manage NOWS symptoms [9]. As not all infants with prenatal opioid exposure will require medication, this extended observation period may not be necessary for all infants.

Few predictive models are available for determining the onset and severity of NOWS. Of the existing models, none have demonstrated the performance necessary for clinical implementation [10, 11]. The NICU Network Neurobehavioral Scale (NNNS), the precursor to the NeoNatal Neurobehavioral Scale II (NNNS-II), has been used extensively in neonatal populations with prenatal opioid exposures [12–16]. The NNNS was developed as part of the Maternal Lifestyle Study to study the effects of prenatal drug exposure, including opiates, on child outcomes [17]. Prior studies using the NNNS have identified neurodevelopmental profiles that describe subgroups of neonates with similar neurobehavioral patterns, or combinations of NNNS summary scores [12–15]. Atypical NNNS profiles have been related to long-term neurodevelopmental outcomes in infants with prenatal opioid exposure [14, 15].

The NNNS-II, administered in this study, is a comprehensive, standardized newborn neurobehavioral assessment. Recent studies using the NNNS-II have shown that neonates with prenatal opioid exposure have distinctive patterns of neurobehavior and that the NNNS-II may predict NOWS severity prior to clinical signs of NOWS [18, 19]. It has yet to be shown whether newborn neurobehavior, especially neurobehavioral profiles, is associated with the need for pharmacological treatment for infants with prenatal opioid exposure. Predicting the need for treatment prior to meeting NOWS symptom thresholds is significant as it could allow for shorter hospital stays, enable infants requiring medication to start treatment sooner, and lead to reduced healthcare costs. The objective of this study was to test whether neonatal neurobehavior in the first two days after birth is associated with the need for pharmacological treatment in infants with prenatal opioid exposure.

## Methods

### Participants

Participants were part of the Child and Family Study, a prospective multi-site study seeking to identify novel clinical predictors of NOWS. 217 mother-infant dyads with prenatal opioid exposure were recruited from prenatal clinics and postnatally at Women and Infant’s Hospital of Rhode Island and University of Utah Hospital between 2019 and 2025. Mothers were approached for consent if prenatal opioid use was identified during pregnancy or at delivery via maternal medical record, a positive maternal urine toxicology during pregnancy or at hospital admission, and/or a positive infant umbilical or urine toxicology screen after birth. Newborns were excluded if they had congenital anomalies, genetic syndromes, metabolic disturbances, sepsis, asphyxia, seizures, respiratory failure, gestational age < 33 weeks, were medically unstable for the NNNS-II exam, unable to take oral medications, or if their caregiver was unable to provide informed consent. Study procedures were approved by the Institutional Review Board of each study site and all participants provided written informed consent for their participation.

### Measures

#### NeoNatal Neurobehavioral Scale – II (NNNS-II)

Trained examiners administered the NNNS-II exam to assess neonatal neurobehavior within 24–48 hours after birth, prior to NOWS treatment. The NNNS-II is a revised and shortened version of the original NNNS exam—a comprehensive standardized evaluation of newborn neurobehavioral performance highlighting neurobehavioral regulation, reflexes, tone and signs of stress and abstinence in the neonate [17]. In the NNNS-II, the original NNNS summary scales were refined and items were retained that statistically contributed to the sensitivity and specificity of the exam. The NNNS-II yields 8 summary scores: attention, handling, self-regulation, arousal, tone, non-optimal reflexes, quality of movement, and stress abstinence. The 15-minute exam was conducted in the infant’s room or in a semi-private room in the nursery.

#### NOWS Treatment

Information about NOWS symptom assessment and pharmacological treatment was abstracted from the infant’s medical record. Initiation of pharmacological treatment for NOWS was based on hospital practices at each site. Women and Infants Hospital used the Finnegan Neonatal Abstinence Scoring Tool (FNAST) [20]. University of Utah Hospital used Neonatal Withdrawal Inventory (NWI) and Eat Sleep Console (ESC) [21, 22]. Infants were assessed every 2–4 hours. The FNAST is arguably the most widely used, quantifiable NOWS assessment tool. Criteria for initiating pharmacological treatment consisted of 3 consecutive scores greater than 7 or 2 consecutive scores greater than 11. The NWI is an 8-item, empirical measure of infant withdrawal derived from the FNAST. Treatment was initiated when the infant received one or more scores of 8 or higher. The ESC tool is a newer assessment focusing on vital infant functions such as eating and sleeping. Criteria for initiating pharmacological treatment were: inability for the infant to eat the appropriate amount based on age, sleep undisturbed for a minimum of 1 hour in between care times, and/or be consoled within 10 minutes [23]. To account for variability in rates of pharmacological treatment based on these different assessments, we adjusted for study site in the analysis.

#### Statistical Analysis

Maternal substance use and maternal and infant characteristics were examined in infants treated versus those not treated for NOWS. Means and standard deviations were used for continuous measures. Categorical variables are expressed as observed counts and percentages. Significance levels for differences between treated and not treated infants were derived from one-way Analysis of Variance (ANOVA) and chi-square tests for continuous and categorical variables, respectively. Latent profile analysis (LPA) was used to classify infants into mutually exclusive neurobehavioral groups based on the 8 NNNS-II summary scores. LPA models with different numbers of profiles were fit and the model containing the optimal number of profiles was identified. Determination of the best model fit was assessed via Bayesian information criteria (BIC) with the smallest BIC value indicating the best fit as well as higher entropy, non-significant bootstrapped likelihood ratio tests and a sufficient number of cases in each profile (> 5% of the sample). Missing data were handled using full information maximum likelihood. To determine if NNNS-II summary scores and NNNS-II profiles were associated with the need for pharmacological treatment, we conducted logistic regression models with treatment as the dependent variable. Adjusted logistic regression models additionally controlled for study site.

## Results

### Descriptive Statistics

**Figure 1:**
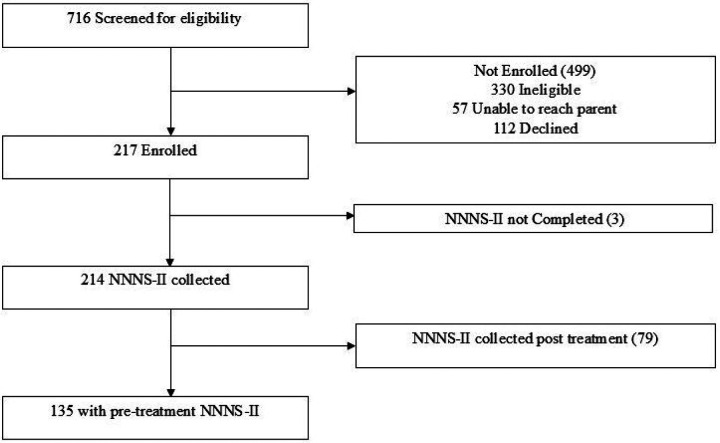
Screening, Eligibility, and NNNS-II Sample Size Consort Diagram. This outlines participant flow from initial contact through enrollment to inclusion in this analysis.

217 mother and newborn dyads from Utah (n = 90) and Rhode Island (n = 127) were enrolled. Of those, 135 had an NNNS-II exam prior to becoming symptomatic for NOWS and are included in this analysis. Symptomatic was defined as at least *one* score of an 8 or higher on the FNAST or NWI, or any *yes* on the ESC. Figure 1 outlines participant flow from initial contact through enrollment to inclusion in this analysis. For this analysis, participants were divided into two groups: those receiving pharmacological treatment (*n* = 37) and those that did not (*n* = 98). Descriptive maternal and infant characteristics, separated by treatment group, are described in Tables 1 and 2. Of note, the mean number of opioids mothers used did not differ between the treatment and no treatment groups (2.2 v 2.1, *p* = 0.67). Polysubstance use (e.g., marijuana, tobacco, and stimulants) was more common in the treatment group (Table 2). 65% of the infants who required treatment had DCYF involvement as compared to 36% in the no treatment group (*p* = 0.002). Additionally, fewer infants in the treatment group (48.6% v 69.4%, *p* = 0.025) were breastfed. Of the 135 participants, 74 received FNAST, 21 received NWI, 24 received ESC, and 16 infants in Utah did not receive a NOWS assessment. None of the infants lacking a NOWS assessment received pharmacological treatment.

### NNNS-II and NOWS Treatment

**Figure 2:**
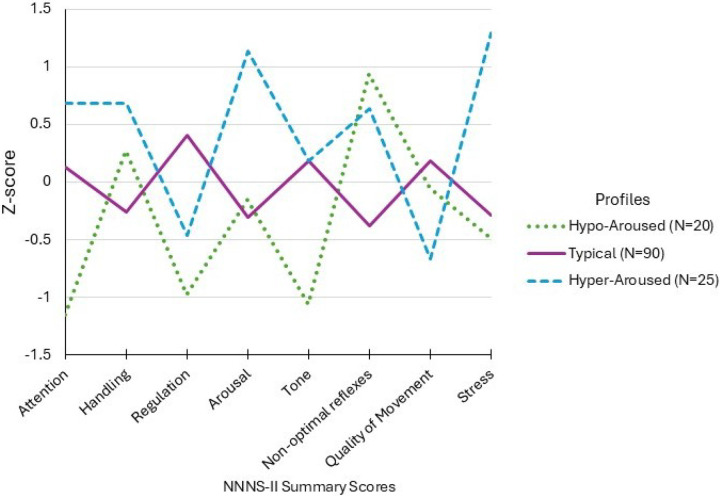
NNNS-II Summary Scores by Latent Profile. LPA analysis yielded three profiles: hyper-aroused (N=25, dashed blue line), hypo-aroused (N=20, dotted green line), and typical (N=25, solid purple line).

We fit LPA models with increasing number of profiles to determine the optimal solution. Based on our selection criteria, the 3-profile solution best fit the NNNS-II data with fit statistics shown in Table 3. Model entropy and average class probabilities were highest for the 3-profile solution and the 4-profile solution failed to converge. Profile 1 included 20 subjects (15%, Fig. 2). These infants showed the lowest attention and regulation, hypotonia, and non-optimal reflexes and were classified as hypo-aroused. Profile 2 comprised of 90 subjects (67%). These infants displayed average NNNS-II scores and were labeled as showing typical neurobehavior. Profile 3 included 25 subjects (19%). This group showed high arousal and stress along with poor quality of movement and were classified as hyper-aroused.

Infants with an atypical NNNS-II profile (either hyper- or hypo-aroused) were more likely to receive pharmacological treatment for NOWS (OR = 3.45, 95% CI 1.21–9.81) compared to infants with the typical profile (*p* < .05, Table 4). Examining individual summary scores, we found that infants with greater non-optimal reflexes were more likely to receive pharmacological treatment for NOWS (OR = 1.55, 95% CI 1.00–2.40) and infants with increased signs of stress and abstinence were also more likely to receive treatment (OR = 3.11, 95% CI 1.16–8.33, all *p’s* < .05). Conversely, infants with better quality of movement were less likely to receive treatment (OR = 0.53, 95% CI 0.33–0.84, *p* < .05).

## Discussion

The overall goal of this study was to investigate whether neonatal neurobehavioral profiles as assessed by the NNNS-II within the first two days of life were associated with the need for pharmacological treatment in infants with prenatal opioid exposure. We found that infants could be classified into one of three distinct phenotypes, a hyper-aroused profile, a typical profile, and a hypo-aroused profile (Fig. 2). Atypical NNNS-II profiles, which included infants that were either hyper- or hypo-aroused, were more likely to require pharmacological treatment for NOWS symptoms. Analysis of NNNS-II summary scores showed that infants with non-optimal reflexes and increased signs of stress and abstinence were more likely to receive pharmacological treatment whereas infants with better quality of movement were less likely to receive treatment. These findings reveal the importance and potential clinical utility of neonatal neurobehavioral phenotypes when predicting the need for pharmacological treatment in infants with prenatal opioid exposure.

NOWS severity and clinical presentation is highly variable and impacted by factors such as polysubstance use, postnatal feeding (e.g., breastfeeding vs formula), and complex social dynamics, consistent with the differences we found between the treatment and no treatment groups [24–26]. Additionally, our study confirms previous findings that infants with more signs of stress and abstinence on the NNNS are more likely to require pharmacological treatment than those who do not [27]. Furthermore, recent studies using the NNNS-II have shown that the NNNS-II summary scores may be associated with NOWS severity including the infant’s length of treatment and number of pharmacological medications required [19]. The Finnegan scoring items have also been shown to be significantly correlated with the NNNS items indicating that the NNNS can be used to supplement existing tools for identification of infants at risk for NOWS [28]. When evaluating infants at risk for NOWS, it is important to consider the whole child. The NNNS-II profiles presented in this study are a holistic characterization of the child’s neurobehavioral repertoire and may have value in shaping the approach to care for infants at risk for NOWS.

Neonatal neurobehavioral profiles have previously been studied in various clinical populations including infants born preterm and infants with prenatal opioid exposure to identify groups of infants with similar neurobehavioral characteristics [13–15, 18, 29]. Profiles provide a way of looking at the whole child with clearer clinical cutoffs as compared to summary scores alone. The two dysregulated profiles seen in this study, hyper- and hypo-aroused, have been previously identified using the NNNS in a study of both term and pre-term infants with different perinatal exposures [29]. In a study of infants with prenatal methadone exposure, infants were classified into four profiles. Infants in profile 4 were characterized by the highest arousal, excitability, hypertonicity, non-optimal reflexes and stress abstinence and those in profile 1 exhibited the lowest attention and highest lethargy [30]. Beyond these populations, similar NNNS phenotypes are evident in low-risk infants with no prenatal substance exposure or other risks for neurobehavioral deficits [31]. Thus, the atypical hyper- and hypo-aroused profiles are commonly observed phenotypes in neonatal populations.

In children with prenatal opioid exposure, atypical NNNS profiles have been associated with a greater likelihood of receiving higher doses of treatment drugs (morphine or methadone), in addition to predicting infant behavioral and developmental outcomes [13–15]. However, these studies used the original version of the NNNS exam. More recently, NNNS-II profiles have been used to compare the neurobehavior of infants with and without prenatal opioid exposure revealing that infants with prenatal exposure to opioids and other psychotropic substances have distinctive patterns of neurobehavior. Four NNNS-II profiles were identified with profile 4 most closely resembling a hyper-aroused phenotype; infants in this profile were more likely to have prenatal opioid exposure compared to those in other profiles [18]. The present study builds upon the prior findings and is the first to show that NNNS-II profiles are associated with the need for pharmacological treatment in infants with prenatal opioid exposure.

The findings of our study in conjunction with the current literature emphasize the clinical utility of the NNNS-II exam when caring for and assessing infants at risk for NOWS. These findings should be interpreted considering the following limitations. Due to a small sample size, the two atypical NNNS-II profiles were grouped together for analysis despite their opposing neurobehavioral characteristics. Future studies with a larger sample size will allow for analysis of the hyper- and hypo-aroused infants separately and therefore could provide improved clinical guidance for these vulnerable infants. Additionally, our cohort includes three different NOWS assessments which play a direct role in infants’ treatment outcomes. However, this is representative of the variability in medical approaches to evaluate and care for infants with prenatal opioid exposure across the U.S. [32, 33]. It is notable that the NNNS-II was associated with the need for pharmacological treatment in our study sample despite the difference in clinical practices across sites.

## Conclusion

In conclusion, neonatal neurobehavior is associated with the need for pharmacological treatment in infants with prenatal opioid exposure. Information about NNNS-II profiles could be used to contribute to an innovative predictive model for determining whether infants with prenatal opioid exposure will require pharmacological treatment for NOWS. Early identification of those infants at risk for more severe NOWS symptoms could enable earlier treatment, potentially reducing length of stay and associated healthcare costs. Additionally, given that studies using the original NNNS have shown predictive validity of NNNS profiles for downstream developmental outcomes and delays, if our profiles are similar, we can hypothesize that infants with atypical profiles will have similar developmental challenges. Further research is needed to understand the predictive properties of each atypical profile, along with the effect of opioid type and other measures of NOWS severity when predicting treatment outcomes.

## Figures and Tables

**Table 1:** Maternal and Infant Characteristics by Infant Pharmacological Treatment Group

	Treatment (N = 37)	No treatment (N = 98)	p-value
	M (SD) or N (%)	M (SD) or N (%)	
**Maternal Characteristics**			
Less than high school degree	7 (19.4%)	24 (25.8%)	0.448
Adequate prenatal care	28 (75.7%)	79 (81.4%)	0.457
No partner	20 (54.1%)	42 (43.3%)	0.264
Hypertension	6 (16.2%)	19 (19.4%)	0.672
Residential Treatment	8 (21.6%)	20 (20.4)	0.877
Inpatient detox during pregnancy	8 (25.0%)	17 (19.1%)	0.480
Bipolar	12 (32.45)	18 (18.8%)	0.091
PTSD	14 (37.8%)	32 (33.3%)	0.625
Hepatitis C	15 (41.7%)	29 (30.5%)	0.228
**Infant Characteristics**			
Sex (Male)	15 (40.5%)	52 (53.1%)	0.194
Gestational Age (weeks)	38.6 (1.3)	38.1 (1.7)	0.132
Birth Weight (grams)	2899 (547)	3014 (546)	0.236
Head circumference at birth (cm)	33.3 (2.0)	33.7 (1.9)	0.291
Length at birth (inches)	47.9 (3.0)	48.5 (2.6)	0.237
DCYF Involvement	24 (64.9%)	35 (35.7%)	0.002
Breastfeeding	18 (48.6%)	68 (69.4%)	0.025
Maximum Finnegan Score	11.6 (1.9)	7.7 (1.9)	< 0.001

*Note*. PTSD = pst-traumatic stress disorder; DCYF = Department of Children, Youth, and Families

**Table 2:** Maternal Substance Use by Infant Pharmacological Treatment Group

	Treatment (N = 37)	No treatment (N = 98)	p-value
	M (SD) or N (%)	M (SD) or N (%)	
**Opioids**			
Number of opioids	2.2 (1.5)	2.1 (1.3)	0.673
Fentanyl	17 (45.9%)	27 (28.1%)	0.050
Buprenorphine	25 (67.6%)	56 (58.3%)	0.328
Methadone	13 (35.1%)	32 (33.3%)	0.844
Heroin	12 (32.4%)	28 (29.2%)	0.713
Morphine	7 (18.9%)	19 (19.8%)	0.909
Oxycodone	4 (10.8%)	19 (19.8%)	0.308
Any illicit opioid	24 (64.9%)	58 (59.2%)	0.547
**Other substances**			
Alcohol	5 (13.5%)	11 (11.2%)	0.714
Marijuana	20 (54.1%)	29 (29.6%)	0.008
Nicotine	34 (91.9%)	66 (67.3%)	0.004
Any stimulant	37 (100%)	74 (75.5%)	< 0.001
Any benzodiazepines	14 (37.8%)	18 (18.4%)	0.018
Any anti-anxiety	22 (59.5%)	43 (44.8%)	0.129
Any anti-depressants	20 (54.1%)	40 (41.7%)	0.198
Any antipsychotic	10 (27.0%)	18 (18.8%)	0.294

**Table 3:** Model Fit Statistics for LPA Models

Number of Profiles	Convergence Problems	Lowest LLH Replicated	BIC	Entropy	Smallest Class Size	LMRT *p*-value	BLRT *p*-value
1	No	Yes	2987.13	--	--	--	--
2	No	Yes	2976.85	0.677	60 (44%)	0.277	< 0.0001
3	No	Yes	2992.55	0.842	20 (15%)	0.137	0.0128
4	Yes	--	--	--	--	--	--

Note: *BIC* sample size-adjusted Bayesian Information Criterion, *BLRT* bootstrapped likelihood ratio test, *LLH* log likelihood, *LMRT* Lo–Mendell–Rubin test, *LPA* latent profile analysis. Convergence problems were noted when the majority of solutions failed to converge.

**Table 4:** NNNS-II Summary Scores and Profiles by Treatment

	Treatment (N = 37)	No treatment (N = 98)	Unadjusted OR (95% CI)	Adjusted OR (95% CI)
**NNNS-II Summary Scores**				
Attention	3.8 (0.9)	4.0 (1.3)	0.89 (0.57, 1.37)	0.84 (0.52, 1.35)
Handling	4.7 (1.8)	4.7 (1.8)	0.99 (0.79, 1.25)	1.06 (0.83, 1.36)
Self-Regulation	4.4 (1.0)	4.4 (1.2)	0.98 (0.69, 1.40)	0.79 (0.52, 1.21)
Arousal	5.1 (2.0)	5.2 (1.6)	0.97 (0.78, 1.21)	1.04 (0.82, 1.33)
Tone	5.2 (0.9)	4.9 (0.7)	1.63 (0.95, 2.77)	1.44 (0.82, 2.54)
Non-optimal Reflexes	4.0 (1.1)	3.8 (1.0)	1.21 (0.82, 1.78)	1.55 (1.001, 2.40)
Quality of Movement	5.8 (1.0)	6.2 (0.9)	0.60 (0.39, 0.93)	0.53 (0.33, 0.84)
Stress Abstinence	2.1 (0.4)	1.9 (0.4)	2.87 (1.11, 7.39)	3.11 (1.16, 8.33)
**NNNS-II Profiles**				
Hyper/Hypo	15 (40.5%)	30 (30.6%)	1.55 (0.71, 3.39)	3.45 (1.21, 9.81)
Typical	22 (59.5%)	68 (69.4%)	-	-

Note: Adjusted OR come from models adjusted for study site. OR = odds ratio, CI = confidence interval, NNNS-II = NeoNatal Neurobehavioral Scale II
